# A Comparative Study Between Use of Topical Honey and Edinburgh University’s Solution of Lime (EUSOL) Dressing in Necrotizing Fascitis Wounds

**DOI:** 10.7759/cureus.33825

**Published:** 2023-01-16

**Authors:** Lajpat Rai, Muhammad Ali Ghufran, Khursheed Ahmed Samo, Munawar Hussain Mangi, Jahanzaib Babar, Mujeeb Rehman Abbasi

**Affiliations:** 1 Surgery, Dow University of Health Sciences, Civil Hospital Karachi, Karachi, PAK; 2 General Surgery, Shaheed Mohtarma Benazir Bhutto Medical College Lyari, Karachi, PAK; 3 Surgery, Dr. Ruth K. M. Pfau Civil Hospital Karachi, Karachi, PAK; 4 Surgery, Dow University of Health Sciences, Karachi, PAK

**Keywords:** necrotizing fasciitis, eusol, honey, randomized trials, necrotizing soft tissue infection

## Abstract

Introduction

Necrotizing soft tissue infection is potentially life-threatening and involves subcutaneous fascial planes, later involving overlying skin and, eventually, underlying muscles. Early diagnosis and prompt treatment are necessary for this disease’s management to avoid significant morbidity and fatality. After resuscitation and optimization, early surgical debridement is followed by serials of dressing. Various materials like Edinburgh University’s solution of lime (EUSOL), normal saline, povidone-iodine, and honey have been used as dressing solutions for necrotizing fasciitis. This study is based on comparing the effects of honey and EUSOL as dressing solutions in necrotizing fasciitis wounds.

Methods

A randomized clinical trial was conducted at the Civil Hospital Karachi, from March 2020 to July 2021. This study has been approved by the ethical review committee of the institution and registered at clinicaltrial.gov. Based on the dressing solution for necrotizing fasciitis, patients were divided into two groups, The honey group (intervention group) contains 90 patients, and the EUSOL group (control group) has 85 patients.

Results

A total of 175 patients’ data were analyzed, 90 in the honey group and 85 in the EUSOL group. Patients presented to the hospital with symptoms of 6.20 ± 2.72 days in the honey group and 6.67 ± 4.08 days in the EUSOL group. The days required for clearance of slough in the honey group were 2.83 ± 0.79, while 2.48 ± 0.82 days in the EUSOL group with a p-value of 0.005. The duration of hospital stay was 4.96 ± 1.31 days in the intervention group and 9.33 ± 1.45 days in the control group, with a p-value of 0.007. Wound healing days were 20.23 ± 4.45 in the intervention group while 28.38 ± 7.06 days in the control group, with a significant p-value of 0.000.

Conclusion

While managing necrotizing soft tissue infection wounds with honey. Honey promotes faster wound healing and shorter hospital stays compared to EUSOL.

## Introduction

The incidence of necrotizing fasciitis (NF) is 0.3 to 5.0 per 100,000 individuals globally [1]. NF/necrotizing soft tissue infection is a potentially life-threatening, quickly progressive soft tissue infection. It involves subcutaneous fascial planes, later involving overlying skin and ultimately, the underlying muscles [2,3]. NF is known as hospital gangrene, streptococcal hemolytic gangrene, synergistic necrotizing cellulitis, flesh-eating disease, and Fournier’s gangrene when it involves the perineum. Management of this disease involves early diagnosis and timely intervention to prevent severe morbidity and mortality. Mortality has improved in past decades due to timely surgical intervention and antibiotics, but it is still around 30% [4]. Its management involves fluid resuscitation, broad-spectrum antibiotics, removal of etiology, extensive emergent debridement, serial dressings, and subsequent reconstruction [4]. The primary role following debridement is intravenous fluids, broad-spectrum or organism-sensitive antibiotics, intensive care support, and dressing [4].

The conventional dressing is done with Edinburgh University’s solution of lime (EUSOL). EUSOL is an antiseptic solution composed of chlorinated lime and boric acid. Removing slough and necrotic tissue from the wound is used as a surgical dressing and promotes wound healing. Honey is also used for wound dressings as a topical agent [5]. Honey is a solution, which is viscous, supersaturated sugar derived from nectar gathered by a honeybee. Honey enhances wound healing by providing a moist environment, antibacterial activity, deodorizing, and decreasing inflammation, edema, and exudation. Honey increases the rate of wound healing by promoting angiogenesis, granulation, and epithelization [6,7]. Honey possesses antibacterial activity against clinically essential organisms like methicillin-resistant *Staphylococcus aureus* (MRSA) and vancomycin-resistant *Escherichia coli*, *Pseudomonas aeruginosa*, and 60 other bacterial species, including aerobes, anaerobes, and even some yeast species such as aspergillus and penicillium [5,7].

The aim of this study is to compare the effects of dressings with honey and EUSOL in the management of NF wounds. The primary outcome was to compare the wound healing time. The secondary outcomes were clearance of slough and length of hospital stay.

## Materials and methods

A prospective, randomized clinical trial was conducted from March 2020 to December 2021 (Clinical Trials Registration ID: NCT04831112) to compare the effects of dressing with EUSOL or honey for managing NF wounds in the emergency department after approval from Ethics Review Committee (IRB-1491/DUHS/Approval/2020). After giving their informed consent, patients were assigned to one of the two groups via the lottery method in a single-blinded procedure. Investigators were blinded to the procedure. The manuscript follows the CONSORT guidelines for all analyses and the randomized trial reporting specified in advance [8].

Inclusion criteria included adults (>18 years) with a diagnosis of NF with the broadest span of wound <20 cm and who underwent surgical debridement followed by dressing were enrolled in the study.

Exclusion criteria included diabetic wounds, traumatic wounds, and patients who expired during the postoperative period.

Intervention

This study included patients with necrotizing soft tissue infection admitted via the emergency department and underwent thorough evaluation, including history, examination, and investigation. Following admission and after the stabilization of vital signs with intravenous fluids and empiric antibiotics, all patients underwent surgical debridement. All patients are given daily routine dressings following debridement. A total of 175 patients enrolled in the study, with 90 in the intervention group (honey) and 85 in the control group (EUSOL). A sample of infected tissue during surgical debridement was taken for pus culture and sensitivity and tissue for histopathology. The wound of all patients healed by secondary intention. The wound was covered with a honey layer of 4 ml per square inch, followed by a dressing covered by Opsite, which was changed every 24 hours. During the dressing change, clearance of slough and appearance of granulation were assessed. Ber Honey (*Ziziphus mauritiana*) was collected from the district of Tharparkar Pakistan and sterilized before being used for dressing. Gamma radiation is used to sterilize the honey and get rid of the bacterium spores known to reside in raw honey.

Data collection

The dataset includes the baseline demographic factors including age, gender, comorbidities including diabetes mellitus, ischemic heart disease, chronic liver disease, chronic kidney disease, hypertension, site involved by disease, duration of symptoms, and smoking. The second section contains outcome factors, including days needed for clearance of slough and appearance of granulation tissue, wound healing time, length of hospital stays, and patient satisfaction.

Statistical analysis

SPSS v.25 was used to analyze the data. Statistics were considered significant for a p-value less than 0.05. Categorical data were analyzed as proportions, while continuous parametric data were analyzed as mean with standard deviation. Chi-square was used to measure categorical variables, whereas continuous parametric variables’ variance was assessed using the student’s t-test.

## Results

**Figure 1:**
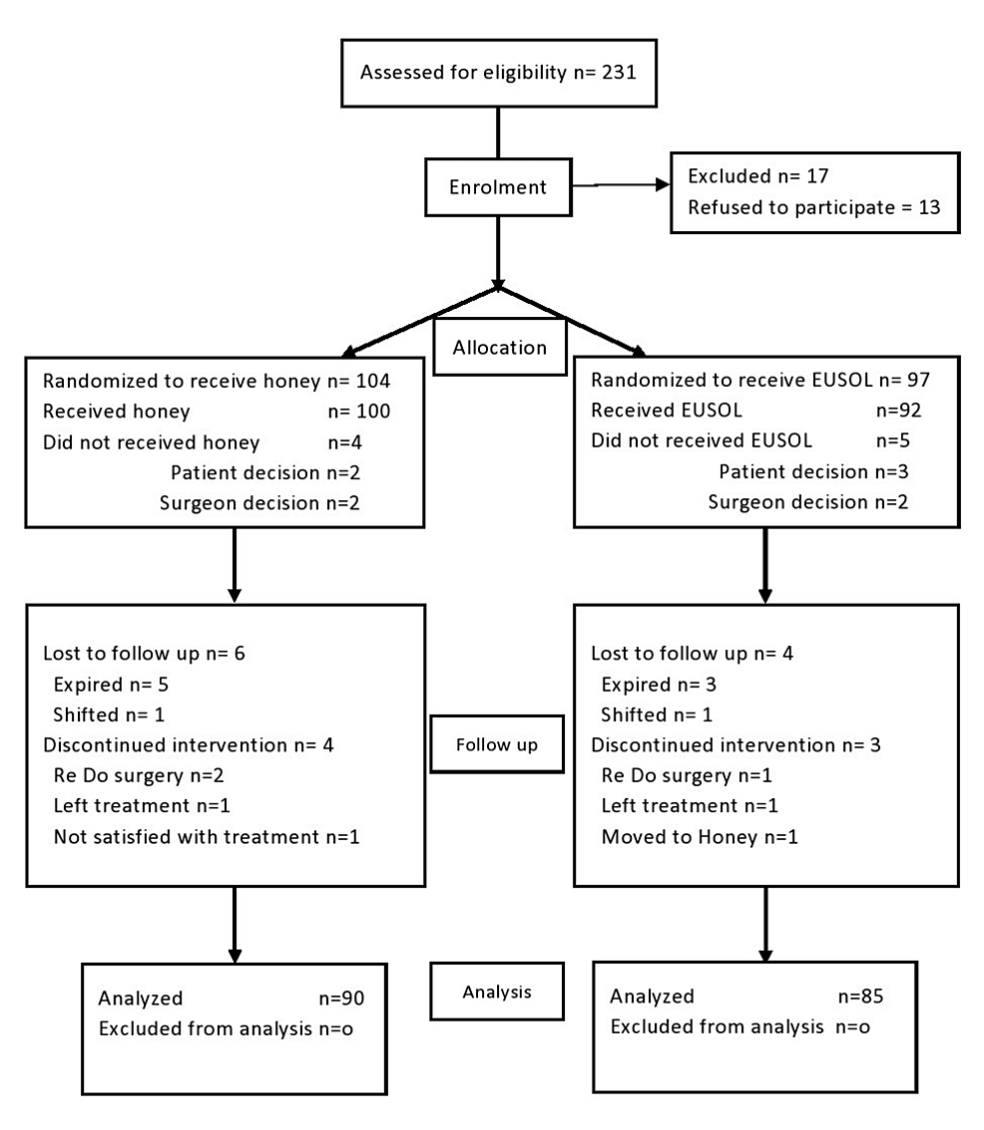
Consort flowchart showing the course of trial EUSOL: Edinburgh University’s solution of lime

A total of 175 patients were analyzed, 90 in the honey group and 85 in the EUSOL group (Figure 1). The mean age in the intervention group was 51.82 ± 12.07 years, while 50.66 ± 13.15 years in the control group. The honey and EUSOL groups had 43 patients with greater than three comorbidities (p=0.76). The lower limb was found to be the most common site of infection, with 57% of patients having lower limb involvement in the honey group and 65 % in the EUSOL group. The results demonstrated a significant difference among the prevalence of smokers in the intervention group (50%) vs. the control group (75%) p=0.001. The mean duration of symptoms before hospital presentation was 6.20 ± 2.72 days in the honey group and 6.67 ± 4.08 days in the EUSOL group, with no statistically significant p-value. For further results, see Table 1.

**Table 1:** Demographic factors (%) DM: diabetes mellitus; IHD: ischemic heart disease; CLD: chronic liver disease; CKD: chronic kidney disease; HTN: hypertension; EUSOL: Edinburgh University’s solution of lime

		Honey	EUSOL	P-value
Age		51.82 ± 12.07	50.66 ± 13.15	0.543
Gender	Male	48(53.3)	36(42.3)	-
	Female	42(46.6)	49(57.6)	
Number of Comorbidities (>3) (DM, IHD, CLD, CKD, HTN)		43(47.7)	43(50.6)	0.76
Site	Lower limb	51(56.6)	55(64.7)	-
	Upper limb	20(22.2)	15(17.6)	
	Trunk	19(21.1)	15(17.6)	
Smoking		45(50)	64(75.2)	0.001
Duration of symptoms (day)		6.20 ± 2.72	6.67 ± 4.08	0.359

The intervention arm showed a reduced number of days in the clearance of the slough (2.83 ± 0.79 days) compared to the control arm (2.48 ± 0.82 days), p=0.005. Healthy granulation tissue is achieved in 7.39 ± 1.48 days in the honey group and 7.05 ± 1.45 days in the EUSOL group with a p-value of 0.126. The mean duration of hospital stay was 4.96 ± 1.31 days in the honey group and 9.33 ± 1.45 days in the EUSOL, with a p-value of 0.007. The satisfaction rate between the two groups did not differ significantly (p=0.226). The intervention group also demonstrated earlier healing time (20.23 ± 4.45) in contrast to the control group (28.38 ± 7.06 days), p=0.000. See Table 2 for further results.

**Table 2:** Outcome factors (%) EUSOL: Edinburgh University’s solution of lime

	Honey	EUSOL	P-value
Day required for clearance of slough	2.83 ± 0.79	2.48 ± 0.82	0.005
Day required for healthy granulation	7.39 ± 1.48	7.05 ± 1.45	0.126
Duration of hospital stay	4.96 ± 1.31	9.33 ± 1.45	0.007
Patient satisfaction	60(66.6)	71(82.3)	0.226
Wound healing time	20.23 ± 4.45	28.38 ± 7.06	0.000

## Discussion

Necrotizing soft tissue infection, including myositis and NF, has a high morbidity and mortality rate. The global burden of disease is 0.3-0.5 per 100,000 [1]. NF can affect any part of the body but is more commonly seen in extremities, specifically lower limbs, usually involving old-age individuals with various comorbidities [9]. NF is classified into two types based on the causative organism. Type 1 NF is a polymicrobial infection of soft tissue and type 2 NF was identified as monomicrobial, both rapidly progressing [9]. Honey was used in this experiment because of the numerous studies demonstrating its positive effects on wound healing as well as its antibacterial and antioxidant characteristics. Additionally, numerous clinical studies with various wound types demonstrated positive outcomes following honey. In a systematic review of 26 trials, including 3011 individuals with acute and chronic wounds, honey improved partial thickness burn healing more quickly than the standard treatment and infected postoperative wounds healed more quickly than with gauze and antiseptics [10].

The results of our study have demonstrated diabetes mellitus to be the most common comorbidity among NF patients. End arteritis resulting from DM, directly and indirectly, decreases the local immune response, consequently increasing the chances of infection. Poor personal hygiene is another predisposing factor, but it is not studied in our study. Poor hygiene acts as a source of microbial entry after itching and scratches [11].

The broad-spectrum antibacterial effect of honey is due to several elements, such as hydrogen peroxide generation, acidity, non-peroxide compounds (such as methyl syringate, defensin 1, and methylglyoxal), and osmotic activity, in addition to encouraging wound healing [6,12]. Honey produces a small amount of hydrogen peroxide, which, when combined with wound exudates, is bactericidal and permanently damages bacterial membranes, proteins, enzymes, and DNA [6]. The pH of honey ranges from 3.2 to 4.5. Its acidity prevents bacterial growth because the ideal pH range for most microbial growth is 7.2-7.4. Additionally, this acidity speeds up oxygen release from hemoglobin, which promotes wound healing [13,14]. As a result of these effects, Honey is effective against a wide range of bacteria and fungi [15]. Honey’s stated minimum inhibitory concentrations (MIC) values were less than 11%. Therefore, honey still has antimicrobial properties even after being diluted by the exudate [14]. There have been no reports of bacterial resistance to honey due to the several antibacterial mechanisms and components operating synergistically [14,15]. Although the initial course of antibiotics was stopped before complete epithelialization without any secondary infections, the multifactorial nature of honey could still account for the wound-healing process in our investigation.

Our study demonstrated a reduction in the number of days for clearance of slough in the honey group compared to the EUSOL group, along with the reduced length of stay in the hospital in the honey group. These findings are in concordance with a study by Ugane et al. in 2018 [11]. In contrast, healthy granulation tissue is achieved earlier in the EUSOL group compared to honey in our observation, which is divergent from the results given by Ugane et al. [11].

The current study proved that honey is effective at speeding up the healing of various NF wounds. This dressing was simple, readily available, did not adhere to the wound bed, and was inexpensive.

Considering its effectiveness and availability in remote areas, honey may be used as an alternative dressing solution to EUSOL in various wound infections. However, large multi-center randomized trials are strongly recommended to validate the efficacy of honey as a dressing solution in superficial and deep soft tissue infections.

Limitations

1) Medical grade honey was not used; 2) the inability to maintain honey’s uniformity; 3) cost-effectiveness not compared with conventional dressing; 4) lack of double-blinded controlled trial on honey dressing because of its distinctive physical characteristics and smell.

## Conclusions

Dressing with honey is superior in terms of early wound healing and decreased length of hospital stay compared to conventional dressing. It is concluded that the topical application of honey promotes wound healing. It is cheaper, more effective, and readily available compared to EUSOL. Therefore, it is recommended to use honey as a dressing solution, especially in the tropics.
